# A combinatorial code for mRNA 3′-UTR-mediated translational control in the mouse oocyte

**DOI:** 10.1093/nar/gky971

**Published:** 2018-10-18

**Authors:** Xing-Xing Dai, Jun-Chao Jiang, Qian-Qian Sha, Yu Jiang, Xiang-Hong Ou, Heng-Yu Fan

**Affiliations:** 1 MOEKey Laboratory for Biosystems Homeostasis & Protection and InnovationCenter for Cell Signaling Network, Life Sciences Institute, Zhejiang University, Hangzhou 310058, China; 2 Fertility Preservation Laboratory, Reproductive Medicine Center, Guangdong Second Provincial General Hospital, Guangzhou 510317, China; 3 Key Laboratory of Reproductive Dysfunction Management of Zhejiang Province, Assisted Reproduction Unit, Department of Obstetrics and Gynecology, Sir Run Run Shaw Hospital, School of Medicine, Zhejiang University, Hangzhou 310016, China

## Abstract

Meiotic maturation of mammalian oocytes depends on the temporally and spatially regulated cytoplasmic polyadenylation and translational activation of maternal mRNAs. Cytoplasmic polyadenylation is controlled by *cis*-elements in the 3′-UTRs of mRNAs including the polyadenylation signal (PAS), which is bound by the cleavage and polyadenylation specificity factor (CPSF) and the cytoplasmic polyadenylation element (CPE), which recruits CPE binding proteins. Using the 3′-UTRs of mouse *Cpeb1, Btg4* and *Cnot6l* mRNAs, we deciphered the combinatorial code that controls developmental stage-specific translation during meiotic maturation: (i) translation of a maternal transcript at the germinal vesicle (GV) stage requires one or more PASs that locate far away from CPEs; (ii) PASs distal and proximal to the 3′-end of the transcripts are equally effective in mediating translation at the GV stage, as long as they are not close to the CPEs; (iii) Both translational repression at the GV stage and activation after germinal vesicle breakdown require at least one CPE adjacent to the PAS; (iv) The numbers and positions of CPEs in relation to PASs within the 3′-UTR of a given transcript determines its repression efficiency in GV oocytes. This study reveals a previously unrecognized non-canonical mechanism by which the proximal PASs mediate 3′-terminal polyadenylation and translation of maternal transcripts.

## INTRODUCTION

The immature oocytes of mammalian species are arrested at the diplotene stage of meiosis-I in growing ovarian follicles (1). The growing oocytes synthesize and store large quantities of dormant mRNAs, which later drive the oocyte’s re-entry into meiosis (2). The resumption of oocyte meiosis is characterized by germinal vesicle breakdown (GVBD), which is followed by two consecutive M-phases (MI and MII) (3). These events of meiotic cell-cycle progression are coupled with transient maternal mRNA polyadenylation, translational activation and then degradation (4,5). The mammalian oocyte maturation process provides a unique and ideal model to study post-transcriptional mRNA regulation, because the fully grown GV stage-arrested oocytes are transcriptionally silent; meiotic maturation and oocyte-to-zygote transition (MZT) are solely driven by the protein products of pre-existing maternal transcripts (6–8).

A key activity driving meiotic progression is provided by the homologous protein kinases ERK1 and 2 (extracellular regulated protein kinase-1 and -2), which induces meiotic spindle assembly and maintains MII arrest (9–11). Recent studies have revealed that a key biochemical function of ERK1 and 2 in oocyte maturation is to couple the mRNA translational activation and mRNA decay with the meiotic cell cycle progression (4,5,12). The most extensively studied mechanism for maintaining repressed maternal mRNAs in GV stage-arrested mouse oocytes and for activating translation during meiotic resumption is mediated by the cytoplasmic polyadenylation element (CPE)-binding protein-1 (CPEB1) (13–16). Cytoplasmic polyadenylation requires two elements in the 3′-untranslated regions (3′-UTRs) of responding mRNAs: the polyadenylation signal (PAS, also known as hexanucleotide AAUAAA), which is bound by the cleavage and polyadenylation specificity factor (CPSF), and the nearby CPE, which recruits CPEBs (16–18). CPEB1 is phosphorylated and activated by ERK1 and 2 as a result of meiotic resumption (4,15,19). In *Xenopus* oocytes, this CPEB phosphorylation increases its affinity for CPSF, which in turn, recruits the cytoplasmic poly(A) polymerase GLD2 (20,21). Nonetheless, studies using *Gld2* knockout mice have concluded that this key poly(A) polymerase in *Xenopus* is dispensable in mouse oocytes, indicating that evolutionally divergent mechanisms are employed (22). Furthermore, the potential involvement of CPSF components in cytoplasmic polyadenylation of mammalian oocytes has never been experimentally tested.

CPE-containing mRNAs display specific translational dynamics during meiotic maturation, suggesting that individual features within their 3′-UTRs determine their response to CPEB1-mediated translational control (23–25). Thus, not all CPE-containing mRNAs are masked in GV stage-arrested oocytes, and the activation of CPE-containing mRNAs does not occur *en masse* at any one time, such as the onset of GVBD. Instead, the polyadenylation of specific mRNAs is temporally regulated (26). Previous studies have reported the composition and regulation of the protein complexes that mediate translational repression and activation of CPE-containing mRNAs in *X*. oocytes (16,27); however, the 3′-UTR features of mammalian genes that define whether an mRNA is a target for CPEB1-mediated translational repression and how the time and extent of cytoplasmic polyadenylation-dependent translational activation is controlled remain unclear.

For example, multiple CPEs are present in the 3′-UTR of *Cpeb1* mRNA (this study), suggesting that CPEB1 controls the translation of its own transcripts. On the other hand, CPEB1 protein is abundantly expressed in GV stage-arrested mouse oocytes, suggesting that *Cpeb1*, as well as some other GV stage-translated transcripts, is exempted from CPE-mediated translational repression (4,28). One of the best studied CPE-containing transcripts, B-cell translocation gene-4 (*Btg4*), encodes a meiotic cell cycle-coupled MZT licensing factor in mammals (5,29,30). *Btg4* mRNA is abundantly expressed in GV-stage mouse oocytes but is kept translationally dormant. The three CPEs and two PASs in the *Btg4* 3′-UTR mediate its translational activation only after GVBD (5,29). It remains unclear why the translational patterns of *Cpeb1* and *Btg4* mRNAs are temporally different, although both contain multiple CPEs and PASs in their 3′-UTRs. *Cnot6l*, one of four genes encoding CCR4–NOT catalytic subunits, is preferentially expressed in mouse oocytes, and mediate meiosis-coupled maternal mRNA decay (Sha *et al*, EMBO Journal 2018, in press). *Cnot6l* was weakly translated in GV oocytes but its translation increased after GVBD (this study). These three genes shared the same *cis*-elements (PASs and CPEs) in their 3′-UTR but with distinct positions. These features suggested that a set of widely applicable combinatory codes determined the translation of these functionally connected transcripts, and tightly linked mRNA translation with key developmental events.

Using the 3′-UTRs of *Cpeb1, Btg4* and *Cnot6l* mRNAs as examples, we analyzed the combinatorial code of CPE and PAS that controls their developmental stage-specific translation during meiotic maturation, which can be potentially used to predict the translational behavior of CPE- and PAS-containing mRNAs in mammalian oocytes.

## MATERIALS AND METHODS

### Mice

Wild-type (WT) Institute of Cancer Research (ICR) mice were obtained from the Zhejiang Academy of Medical Science, China. Mice were maintained under specific-pathogen-free conditions in a controlled environment of 20–22°C, with a 12/12 h light/dark cycle, 50–70% humidity and food and water provided *ad libitum*. Animal care and experimental procedures were conducted in accordance with the Animal Research Committee guidelines of Zhejiang University.

### Oocyte isolation and culture

Three-week-old WT female mice were intraperitoneally injected with 5 IU of pregnant mare serum gonadotropin and humanely euthanized 44 h later. Fully grown oocytes at the GV stage were harvested in M2 medium (M7167; Sigma-Aldrich) and cultured in mini-drops of M16 medium (M7292; Sigma-Aldrich) covered with mineral oil (M5310; Sigma-Aldrich) at 37°C in a 5% CO_2_ atmosphere.

### *In vitro* transcription and preparation of mRNAs for microinjections

To prepare mRNAs for microinjection, expression vectors (pRK5, BD Biosciences) were linearized and subjected to phenol/chloroform extraction and ethanol precipitation. The linearized DNAs were *in vitro*-transcribed using the SP6 mMESSAGE mMACHINE Kit (Invitrogen, AM1450) following the manufacturer’s instructions. The control mCherry mRNA was *in vitro*-polyadenylated using the Poly(A) Tailing Kit (Invitrogen, AM1350). The mRNAs were recovered by lithium chloride precipitation and resuspended in nuclease-free water. The concentration of all injected RNAs were adjusted to 500 ng/μl.

### Microinjection of oocytes

For microinjection, fully grown GV oocytes were harvested in M2 medium with 2 μM milrinone to inhibit spontaneous GVBD. All injections were performed using an Eppendorf TransferMan NK2 micromanipulator. Denuded oocytes were injected with 5–10 pl samples per oocyte. After injection, oocytes were washed and cultured in M16 medium plus 2 μM milrinone at 37°C with 5% CO_2_.

### Ribonucleoprotein immunoprecipitation (RIP) assay

The ribonucleoprotein immunoprecipitation (RIP) assay procedure was modified from previously described (31,32). In brief, oocytes were lysed in lysis buffer (50 mM Tris–HCl [pH 7.4], 1% Triton X-100, 150 mM NaCl, 5 mM ethylenediaminetetraacetic acid (EDTA), protease inhibitor cocktail and RNase inhibitor). After centrifugation, the supernatant was subjected to immunoprecipitation with affinity gels conjugated with the indicated antibodies (Sigma). After incubation at 4°C for 4 h, beads were thoroughly washed with washing buffer (50 mM Tris–HCl [pH 7.4], 0.1% Triton X-100, 500 mM NaCl, 5 mM EDTA, protease inhibitor cocktail and RNase inhibitor). RNA bound to beads was extracted using the RNeasy Mini kit (Qiagen, 74106) according to the manufacturer’s instructions, and were reverse-transcribed with Moloney Murine Leukemia Virus (M-MLV) (Invitrogen). The relative abundance of cDNA was analyzed through quantitative polymerase chain reaction (qPCR). Primer sequences were provided in the Supplementary Table S1.

### Nested Poly(A) tail (N-PAT) assay

Total RNA was isolated from 100 oocytes at the indicated stages using the RNeasy Mini kit (Qiagen, 74106). R1 (5′-GCGAGCTCCGCGGCCGCGT_12_–3′) was anchored to Oligo(dT) by T4 DNA ligase (33,34). Reverse transcription was performed using the SuperScript IV (Invitrogen) with Oligo(dT) anchored R1. The products were used in a PCR reaction with gene-specific primers (Supplementary Table S1) and the dT anchor primer R1. To avoid amplification of poly(A) tails from endogenous transcripts, the first PCR was performed using GFP-specific and PAT anchor primers (18 cycles). To obtain shorter PCR products containing the poly(A) tail, 20% of the product of the first PCR reaction was used as a template for the second PCR using 3′-UTR-specific and PAT anchor primers (20 cycles). The PCR conditions were as follows: 30 s at 94°C, 20 s at 58°C and 40 s at 72°C. The polyadenylation states of the PCR products were analyzed on a 1.5% agarose gel, and images were captured during exposure to ultraviolet light. Signals were quantified using the ‘Plot profiles’ function of the ImageJ software (http://imagej.nih.gov/ij/). Quantified values were normalized by the maximum signal intensity in each lane, and averaged values of three biological replicate were plotted.

### Real-time RT-PCR

Total RNA was extracted using the RNeasy Mini kit (QIAGEN) according to the manufacturer’s instruction, followed by RT using Superscript RT kit (Bio-Rad). The qRT-PCR was performed using a Power SYBR Green PCR Master Mix (Applied Biosystems, Life Technologies) with ABI 7500 RealTime PCR system (Applied Biosystems) using primers listed in Supplementary Table S1.

### Immunofluorescence and confocal microscopy

Oocytes were fixed with 4% paraformaldehyde in phosphate-buffered saline (PBS). They were then permeabilized with 0.3% Triton X-100 in PBS. Antibody staining of CPSF4 was performed using standard protocols described previously (35). Imaging was performed on a Zeiss LSM710 confocal microscope. Semi-quantitative analysis of the fluorescence signals was conducted using the NIH Image analysis program ImageJ, as previously described (36).

### Western blot analysis

Oocytes were lysed in protein loading buffer and heated at 95°C for 5 min. Sodium dodecyl sulphate-polyacrylamide gel electrophoresis and immunoblots were performed following standard procedures using a Mini-PROTEAN Tetra Cell System (Bio-Rad, Hercules, CA, USA). The primary antibodies and dilution factors used are listed in Supplementary Table S2.

### Statistical analysis

Results are given as means ± SEM. All experiments included at least three biological repeats. Results for two experimental groups were compared by two-tailed unpaired Student’s *t*-tests. Statistically significant values of *P* < 0.05, *P* < 0.01 and *P* < 0.001 by two-tailed Student’s *t*-test are indicated by asterisks (*), (**) and (***) respectively. ‘n.s.’ indicates non-significant.

## RESULTS

### Both the proximal and distal PASs mediate the translation activity of *Cpeb1* 3′-UTR at the GV stage

**Figure 1.**
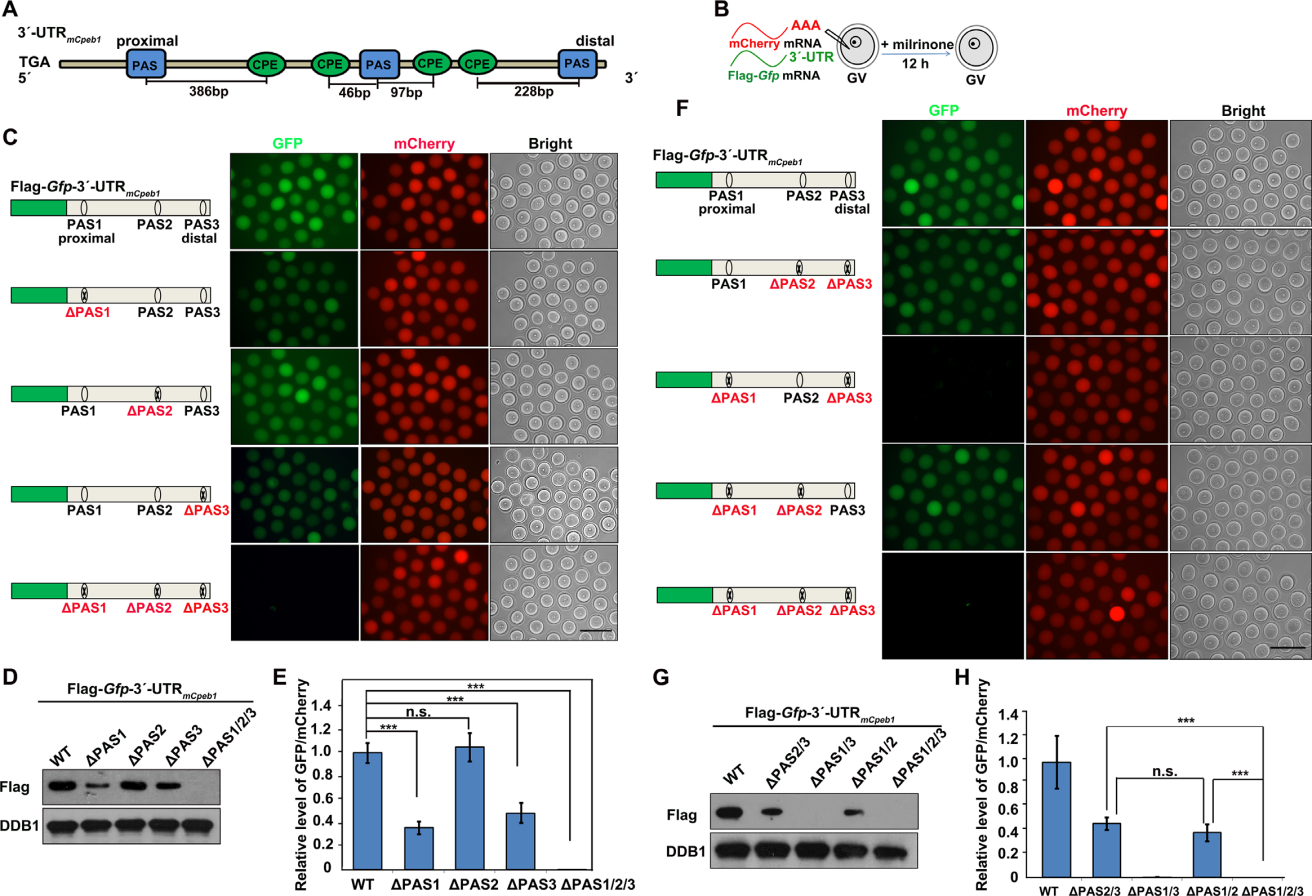
Contributions of PASs to the translational activity of *Cpeb1* 3′-UTR in GV oocytes. (**A**) Schematic representation of the 3′-UTR of mouse *Cpeb1* mRNA. Relative positions of PASs and CPEs are indicated. (**B**) Illustration of mRNA microinjection and oocyte culture in (**C**–**H**). (C and D) Fluorescence microscopy (C) and western blotting (D) results showing the expression of Flag-GFP fused with *Cpeb1* 3′-UTR or its PAS-mutated (ΔPAS) forms. Endogenous DDB1 was used as a loading control. Total proteins from 60 oocytes were loaded in each lane. Scale bar, 100 μm for all images. (E) Relative fluorescence intensity of GFP relative to mCherry in (C). Statistically significant values of *P* < 0.05, *P* < 0.01 and *P* < 0.001 by two-tailed Student's *t*-test are indicated by asterisks (*), (**) and (***), respectively. (F and G) Fluorescence microscopy (F) and western blotting (G) results showing expression level of Flag-GFP after two to three PAS mutations in the *Cpeb1* 3′-UTR. (H) Relative fluorescence intensity of GFP relative to mCherry in (F).

As predicted by an online program (http://genome.crg.es/CPE/server.html), the 3′-UTR of mouse *Cpeb1* contains three putative PASs and four CPEs (Figure 1A). To investigate the combinatorial contribution of these elements to the translation of *Cpeb1* mRNA in GV stage-arrested mouse oocytes, we cloned the mouse *Cpeb1* 3′-UTR (3′-UTR_m_*_Cpeb1_*) and ligated it into the pRK5-Flag-*Gfp* vector, which contains a SP6 transcription initiation sequence. We then *in vitro*-transcribed the unpolyadenylated mRNA encoding Flag-*Gfp*-3′-UTR_m_*_Cpeb1_* and microinjected it into GV stage-arrested oocytes. Therefore, the translational activity of this mRNA depends on 3′-UTR_m_*_Cpeb1_*-mediated *de novo* cytoplasmic polyadenylation. As a control of background translation activity, an *in vitro-*transcribed and *in vitro*-polyadenylated mRNA encoding *mCherry* cDNA was co-injected. Microinjected oocytes were further cultured for 12 h in medium containing 2 μM milrinone that inhibits meiotic resumption (Figure 1B).

Expression of Flag-GFP protein were detected in oocytes by both epifluorescence and western blot (Figure 1C and D). The translation activity of 3′-UTR_m_*_Cpeb1_* was quantified by comparing the GFP and mCherry fluorescence intensities within the same oocyte (Figure 1E). These results indicated that the 3′-UTR of *Cpeb1* mRNA was capable of mediating translation at the GV stage. This was consistent with previous reports where endogenous CPEB1 proteins were present in GV oocytes (4,14). Next, we mutated all three PASs (AATAAA to AAGGAA) in the *Cpeb1* 3′-UTR, and observed that the resulting mRNA was no longer translated in GV oocytes (Figure 1C–E), indicating that PASs in the *Cpeb1* 3′-UTR were required for its translation activity.

Next, we examined the contribution of individual PAS to the translational activity of *Cpeb1* 3′-UTR at the GV stage. According to a previously accepted model, the PAS at the distal end of the 3′-UTR was responsible for cytoplasmic polyadenylation and translation (37,38). Therefore, we mutated the distal PAS in the 3′-UTR of *Cpeb1* and microinjected the *in vitro*-transcribed mRNA into GV oocytes. Unexpectedly, the PAS3-mutated 3′-UTR_m_*_Cpeb1_* was still translated in GV oocytes, although the levels of translated proteins were slightly decreased compared to that of the WT form (Figure 1C–E). The translational levels decreased more significantly when PAS1 was mutated, but remained unaffected after the PAS2 mutation (Figure 1C–E). Furthermore, PAS1 or PAS3 alone is sufficient to maintain 40–50% of the translational activity of *Cpeb1* 3′-UTR in GV oocytes (Figure 1F–H). In contrast, the *Cpeb1* 3′-UTR failed to support translation at the GV stage when only PAS2 was present (Figure 1F–H). Taken together, these results indicated that both the proximal and distal PASs, but not the middle PAS, mediate the translational activity of *Cpeb1* 3′-UTR in meiotic prophase-arrested mouse oocytes. This is an unexpected observation because according to the canonical working model, PASs can only mediate polyadenylation and translational activation when they locate at the distal end of a given transcript (25,38).

### PASs in the proximal 3′-UTR were able to direct cytoplasmic polyadenylation of transcripts

**Figure 2.**
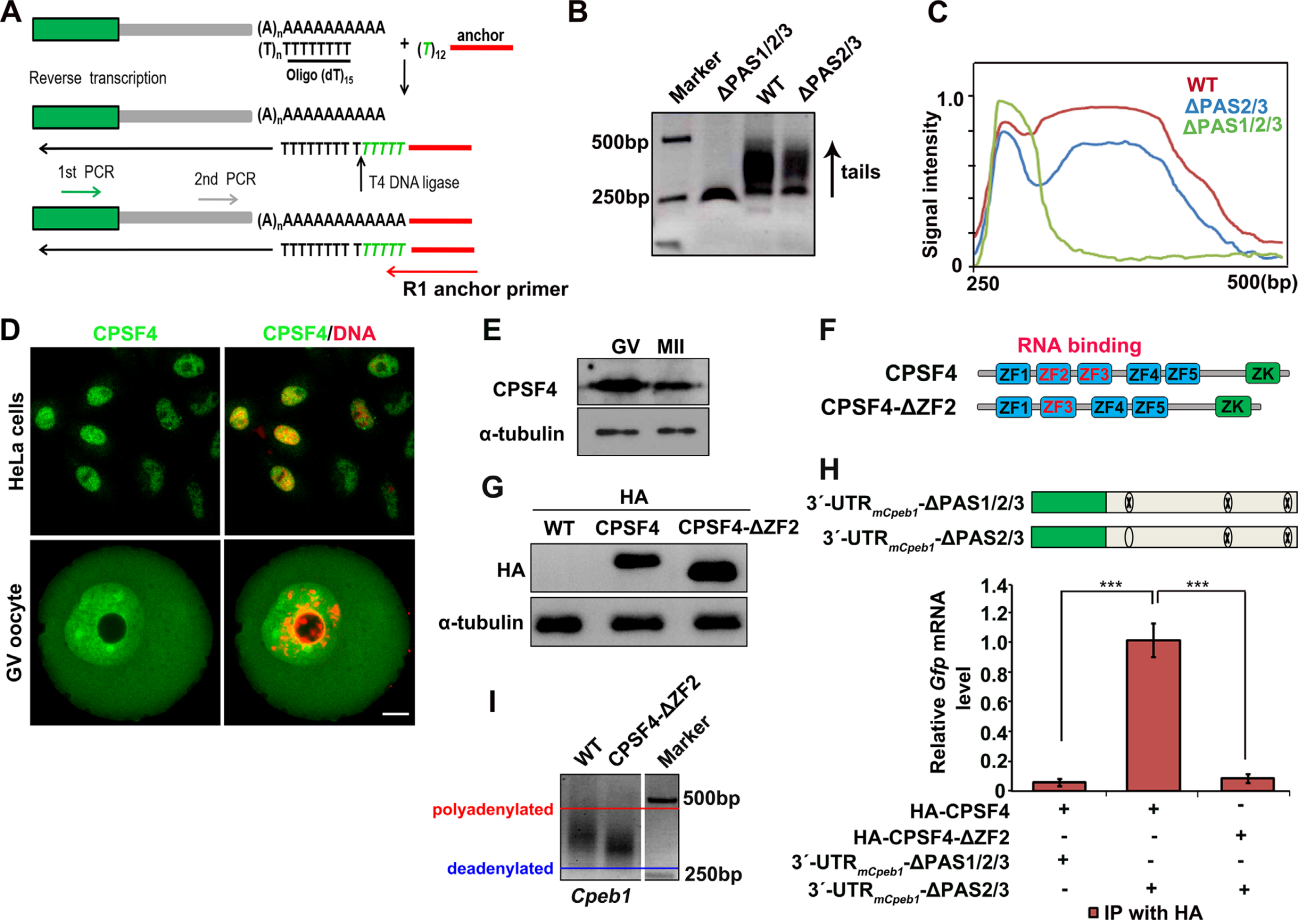
Cytoplasmic polyadenylation of *Cpeb1* 3′-UTR driven by the proximal PAS and CPSF4. (**A**) A schematic representation of the nested PAT (N-PAT) assay. (**B**) Results of the N-PAT assay showing poly(A) tail lengths of the indicated transcripts with different *Cpeb1* 3′-UTRs being microinjected into oocytes at the GV stage. PCR conditions were described in detail in the ‘Materials and Methods’ section. (**C**) Quantification of the PAT assay results in (B). The plots showed the averaged relative signal intensity (*y*-axis) and the length of the PCR products based on mobility (*x*-axis). (**D**) Immunofluorescence results showing localization of endogenous CPSF4 in HeLa cells and GV oocytes. Scale bar, 10 μm. (**E**) Western blot results showing the expression levels of CPSF4 in GV and MII oocytes. α-Tubulin was used as a loading control. Total proteins from 100 oocytes were loaded in each lane. (**F**) Schematic representation of functional domains of mouse CPSF4 and CPSF4-ΔZF2. (**G**) Western blot results showing the expression levels of HA-tagged CPSF4 and CPSF4-ΔZF2 in GV oocytes at 12 h after mRNA microinjection. (**H**) RIP assay results using an HA antibody showing the interactions between CPSF4 (HA-CPSF4 or HA-CPSF4-ΔZF2) and indicated transcripts in GV oocytes. ***, *P* < 0.001 by two-tailed Student's *t-*tests. Levels of *Gfp* mRNA co-precipitated with CPSF4 was detected by quantitative RT-PCR. (**I**) Results of PAT assay showing poly(A) tail lengths of the endogenous *Cpeb1* transcripts in GV oocytes or those microinjected with mRNAs encoding CPSF4-ΔZF2.

Next, we investigated how PAS1 on the proximal region of *Cpeb1* 3′-UTR mediated translation in GV oocytes. Using a modified nested poly(A) tail (N-PAT) assay (Figure 2A), we detected the polyadenylation levels of microinjected Flag-*Gfp*-3′-UTR_m_*_Cpeb1_* transcripts. The exogenous transcripts containing a WT *Cpeb1* 3′-UTR were polyadenylated (Figure 2B and C). As a negative control, mutant forms of the three PASs (ΔPAS1/2/3) abolished the polyadenylation of these transcripts (Figure 2B and C), confirming the specificity of our N-PAT readout. Notably, the Flag-*Gfp*-3′-UTR_m_*_Cpeb1_* transcripts that only have the proximal PAS1 (ΔPAS2/3) were also polyadenylated (Figure 2B and C). Because only the terminal fragments of the exogenous Flag-*Gfp*-3′-UTR_m_*_Cpeb1_* were amplified in the N-PAT assay, this result rules out the possibility that PAS1-mediated terminal polyadenylation in prematurely terminated Flag-*Gfp*-3′-UTR_m_*_Cpeb1_* transcripts. The proximal PAS-mediated polyadenylation of the mRNA tail has not been previously described. Therefore we further investigated the biochemical mechanism underlying this process.

### CPSF4 was involved in the proximal PAS-mediated cytoplasmic translation in oocytes

The CPSF complex plays a key role in nuclear polyadenylation of somatic cells (38). CPSF4 has been recently recognized as a PAS-binding subunit of the CPSF complex (37). While endogenous CPSF4 proteins were exclusively located in the nucleus of HeLa cells, they were present in both the GVs and the ooplasm of fully grown oocytes (Figure 2D), suggesting that CPSF may also participate in the cytoplasmic polyadenylation of oocyte transcripts. WT results also showed that CPSF4 was expressed in mouse oocytes at both GV and MII stages (Figure 2E).

Next, we examined the interaction between *Cpeb1* 3′-UTR and CPSF4 by an RIP assay. CPSF4 contains five zinc finger (ZF) domains and a zinc knuckle (ZK) domain. Among these, ZF2 and ZF3 are responsible for the recognition and binding of PAS (Figure 2F) (37). Because the commercially available CPSF4 antibody did not work well for immunoprecipitation, we expressed a HA-tagged CPSF4 or its ZF2-deleted form (ΔZF2) in GV oocytes by mRNA microinjection. The expression of these proteins in oocytes were confirmed by western blotting (Figure 2G). RIP assay results indicated that the *Cpeb1* 3′-UTR transcripts containing PAS1 (Flag-*Gfp*-3′-UTR_m_*_Cpeb1_*-ΔPAS2/3) co-injected with mRNAs encoding HA-CPSF4 into GV oocytes were enriched in CPSF4 precipitates (Figure 2H). As a negative control, the same transcripts were not precipitated by HA-CPSF4-ΔZF2. Furthermore, mutations in all of the PASs (ΔPAS1/2/3) abolished the interaction between *Cpeb1* 3′-UTR and CPSF4.

Because CPSF4-ΔZF2 fails to bind with PAS, it could function as a dominant negative mutant form to block endogenous CPSF activity in oocytes. CPSF4-ΔZF2 overexpression caused shortening of the poly(A) tail of the *Cpeb1* 3′-UTR transcripts in GV oocytes (Figure 2I), and blocked meiotic resumption-triggered polyadenylation as well as translation of maternal transcripts (Supplementary Figure S1A and B). Phenotypically, overexpression of CPSF4-ΔZF2 had marginal influence on GVBD but significantly blocked PB1 emission and impaired the assembly of a normal meiotic spindle in oocytes (Supplementary Figure S1C–F). Collectively these results suggest that normal CPSF function was required for cytoplasmic polyadenylation of maternal mRNAs in oocytes as well as meiotic cell-cycle progression.

### CPEs repressed the translation of transcripts in GV oocytes by inhibiting PASs that were nearby

**Figure 3.**
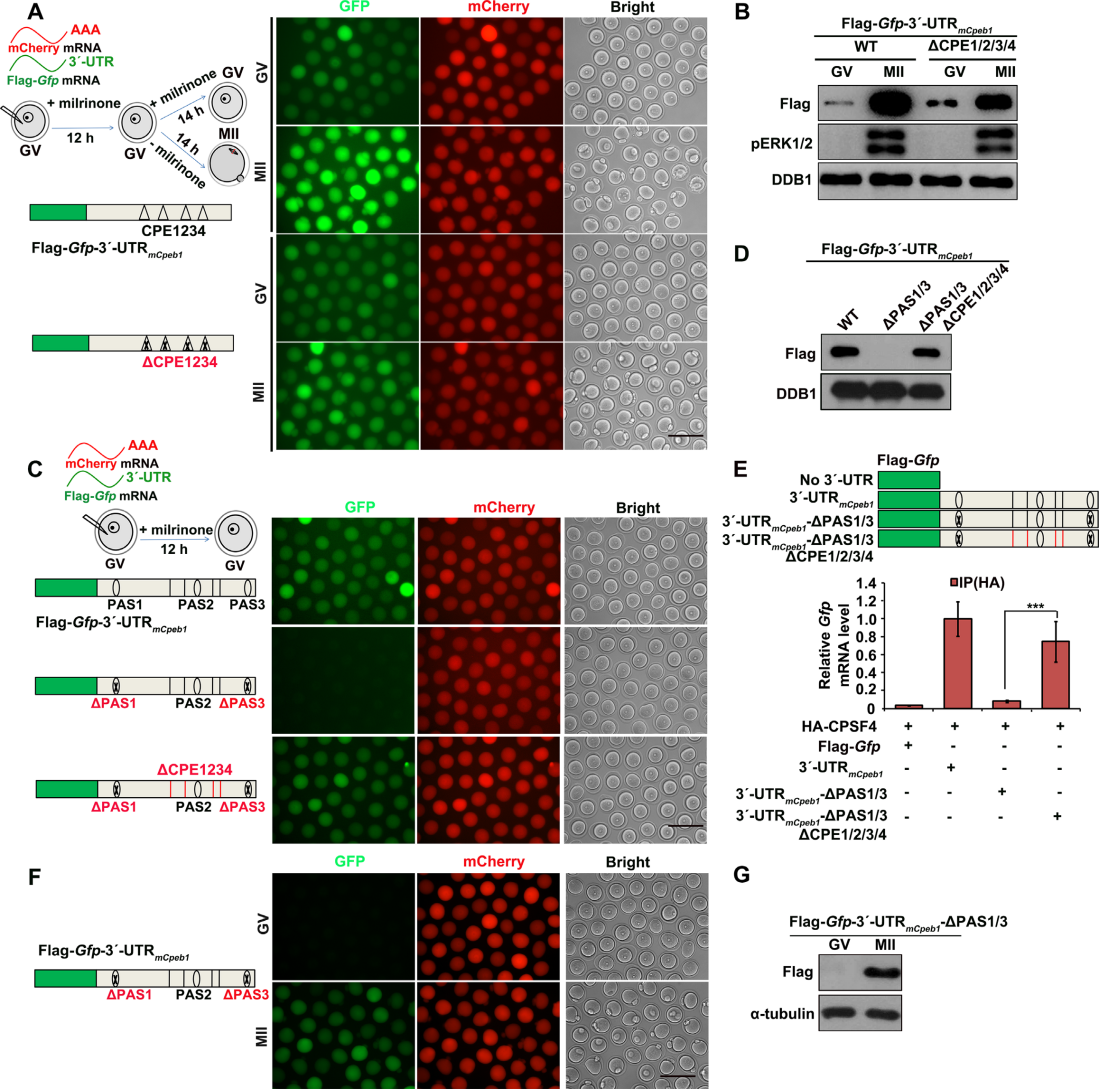
Role of CPEs in regulating the meiotic maturation-coupled *Cpeb1* translation. (**A** and **B**) Fluorescence microscopy (A) and western blot (B) results showing the translational levels of Flag-GFP driven by *Cpeb1* 3′-UTRs with or without CPE mutations, in oocytes arrested at the GV stage and those developed to the MII stage. DDB1 was used as a loading control, and phosphorylated ERK1/2 (pERK1/2) was used to indicate the meiotic cell-cycle stages. Total proteins from 60 oocytes were loaded in each lane. Scale bar, 100 μm. (**C** and **D**) Fluorescent microscopy (C) and western blot (D) results showing the expression level of Flag-GFP driven by *Cpeb1* 3′-UTR with combined PAS and CPE mutations. (**E**) Results of RIP assay with an anti-HA antibody showing the interaction of CPSF4 with indicated transcripts in GV oocytes. ***, *P* < 0.01 by two-tailed Student's *t*-tests. (**F** and **G**) Fluorescence microscopy (F) and western blot (G) results showing translational levels of Flag-GFP driven by *Cpeb1* 3′-UTRs containing only PAS2, in oocytes arrested at the GV stage and those developed to the MII stage. Oocytes were experimentally manipulated as illustrated in (A). α-Tubulin was used as a loading control. Total proteins from 60 oocytes were loaded in each lane. Scale bar, 100 μm.

Next, we investigated why PAS2 in the *Cpeb1* 3′-UTR was incapable of mediating translation in GV oocytes. While PAS2 was flanked by four CPEs, PAS1 and PAS3 were 386 and 228 bp away from the closest CPE, respectively (Figure 1A). Although the Flag-*Gfp*-3′-UTR_m_*_Cpeb1_* transcripts were able to be translated after microinjection into the GV oocytes, a significant increase of translation was observed when the microinjected oocytes were released from the meiotic arrest by milrinone removal (Figure 3A and B). On the other band, mutations of 4 CPEs in *Cpeb1* 3′-UTR caused a slight increase of translational activity at the GV stage and a decrease of translation at the MII stage (Figure 3A and B). This observation confirmed previous findings in *X*. oocytes where CPEs have a bidirectional regulatory effect on cytoplasmic polyadenylation in mouse oocytes: they inhibit mRNA translation at the GV stage, and stimulate translation after meiotic resumption.

More specifically, when we mutated four CPEs in the *Cpeb1* 3′-UTR that only contained PAS2 (3′-UTR_m_*_Cpeb1_*-ΔPAS1/3, which does not support translation in GV oocytes), translational activity was restored (Figure 3C and D). Furthermore, results of the RIP assay indicated that CPSF4 did not bind with PAS2 of the *Cpeb1* 3′-UTR in GV stage-arrested oocytes (Figure 3E). Nonetheless, when we mutated the four CPEs flanking the PAS2, binding between PAS2 and CPSF4 was detected (Figure 3E).

We also analyzed whether the inhibitory effect of CPEs on PAS2 was affected by meiotic resumption. Results of the mRNA microinjection experiment showed that the Flag-*Gfp*-3′-UTR_m_*_Cpeb1_*-ΔPAS1/3 transcripts were translationally dormant in GV oocytes, but were activated when the oocytes were released into meiotic maturation (Figure 3F and G). Therefore, while PAS2 was inhibited by the CPEs flanking it, the translation repressing effect of these CPEs were relieved following meiotic cell-cycle progression.

**Figure 4.**
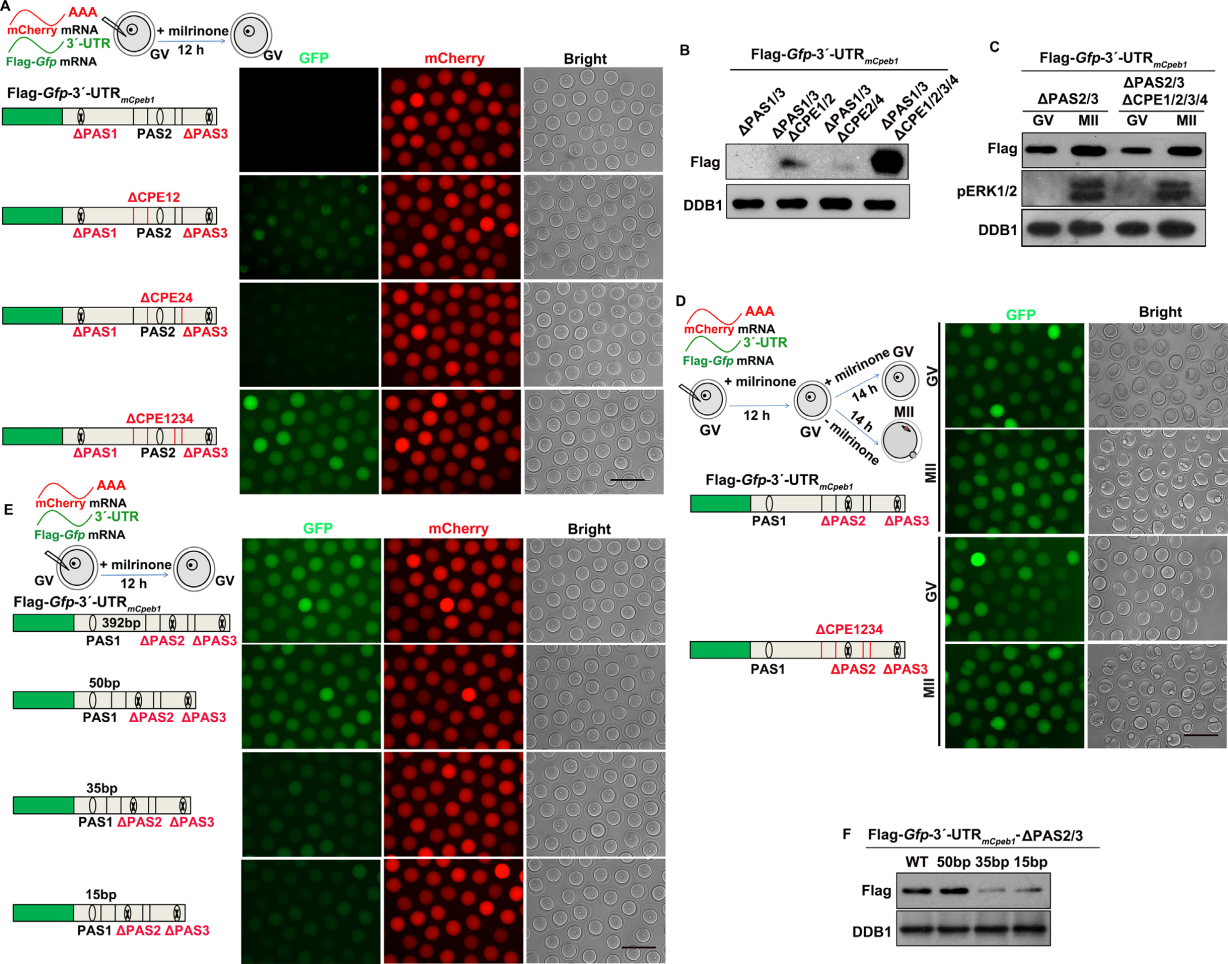
Association of CPEs localization with its translation-repressing effect. (**A** and **B**) Fluorescent microscopy (A) and western blot (B) results showing translation levels of Flag-GFP in GV oocytes, driven by *Cpeb1* 3′-UTR containing combined PAS and CPE mutations as indicated. DDB1 was used as a loading control. Total proteins from 60 oocytes were loaded in each lane. Scale bar, 100 μm. (**C** and**D**) Western blot (C) and fluorescent microscopy (D) results showing translational levels of Flag-GFP driven by *Cpeb1* 3′-UTR containing combined PAS and CPE mutations as indicated, in oocytes arrested at the GV stage and those developed to the MII stage. DDB1 was used as a loading control, and phosphorylated ERK1/2 (pERK1/2) was used to indicate MII arrest. Total proteins from 60 oocytes were loaded in each lane. Scale bar, 100 μm. (**E** and **F**) Fluorescence microscopy (E) and western blot (F) results showing translational levels of Flag-GFP in GV oocytes, driven by *Cpeb1* 3′-UTR with the indicated mutations.

**Figure 5.**
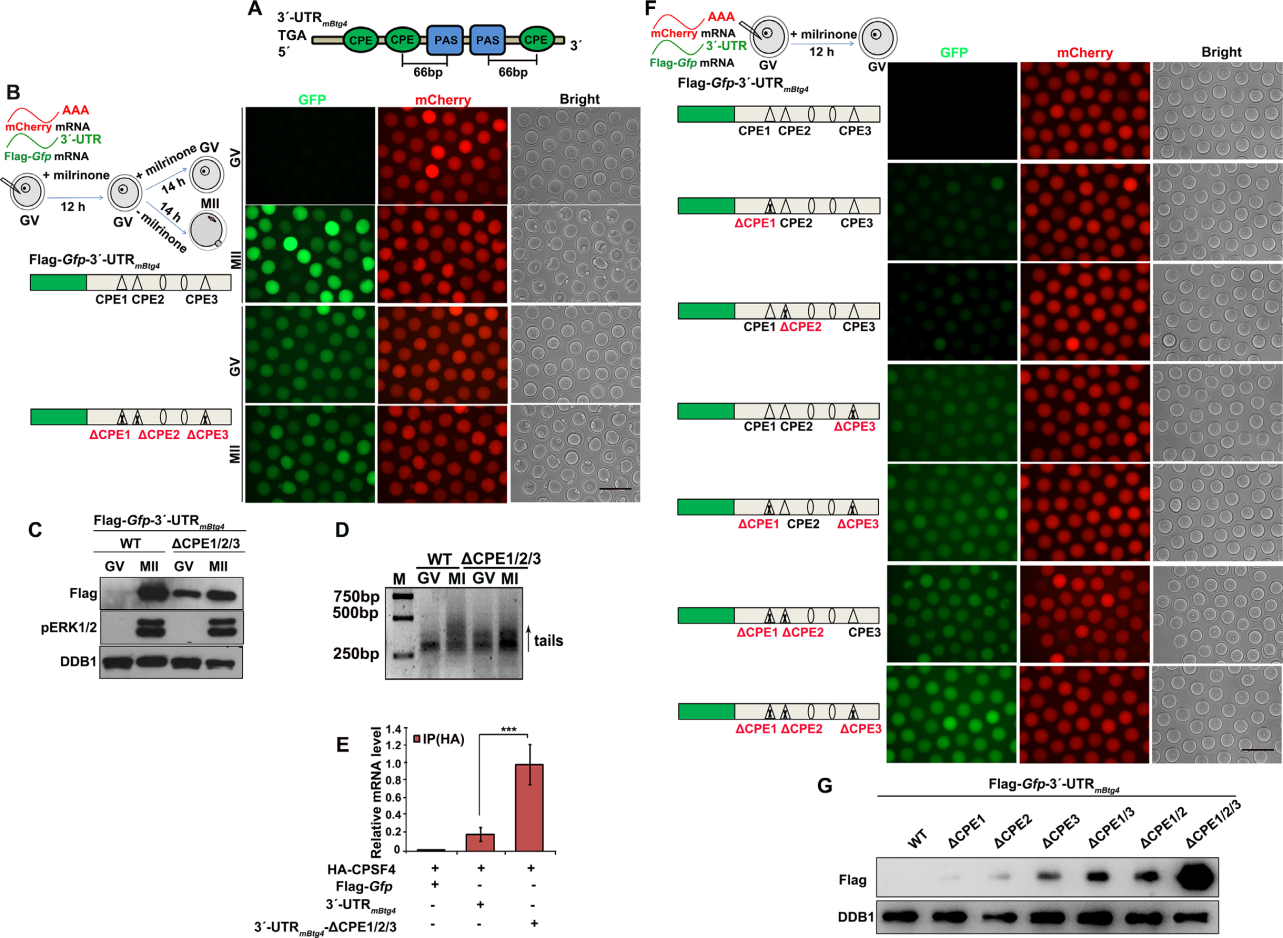
CPEs within the *Btg4* 3′-UTR are required for translation inhibition at the GV stage. (**A**) Schematic representation of the PASs and CPEs in the 3′-UTR of mouse *Btg4* mRNA. (**B** and **C**) Fluorescence microscopy (B) and western blot (C) results showing translational levels of Flag-GFP driven by *Btg4* 3′-UTR with or without CPE mutations, in oocytes arrested at the GV stage and those developed to the MII stage. DDB1 was used as a loading control, and pERK1/2 was used to indicate MII arrest. Total proteins from 60 oocytes were loaded in each lane. Scale bar, 100 μm. (**D**) Results of the N-PAT assay showing polyadenylation of Flag-*Gfp*-3′-UTR_m_*_Btg4_* transcripts with or without the indicated CPE mutations. (**E**) Results of RIP assay showing the interaction between CPSF4 and transcripts containing WT or CPE-mutated *Btg4* 3′-UTR in GV oocytes. ***: *P* < 0.01 by two-tailed Student's *t*-tests. (**F** and **G**) Fluorescence microscopy (F) and western blot (G) results showing translation levels of Flag-GFP in GV oocytes driven by *Btg4* 3′-UTR with or without the indicated CPE mutations.

**Figure 6.**
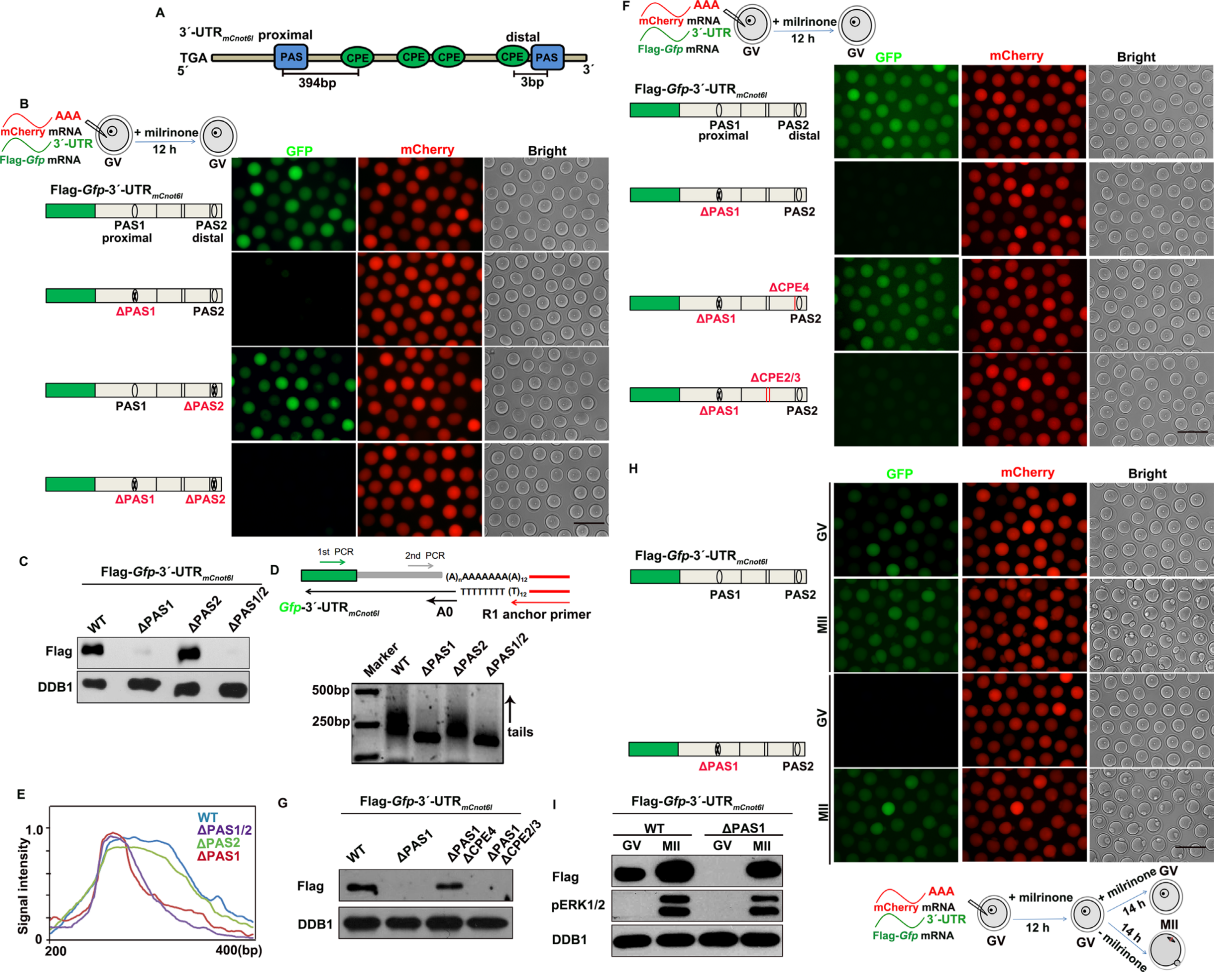
Regulation of meiotic cell cycle-coupled *Cnot6l* 3′-UTR activity by PASs and CPEs. (**A**) Schematic representation of the PASs and CPEs in the 3′-UTR of mouse *Cnot6l* mRNA. (**B** and **C**) Fluorescence microscopy (B) and western blot (C) results showing the translational levels of Flag-GFP driven by *Cnot6l* 3′-UTRs without or with PAS mutations in GV oocytes. (**D**) Results of the N-PAT assay showing polyadenylation of Flag-*Gfp*-3′-UTR_m_*_Cnot6l_* transcripts without or with indicated PAS mutations. (**E**) Quantification of the N-PAT assays in (D). ( **F** and **G**) Fluorescence microscopy (F) and western blot (G) results showing the translational levels of Flag-GFP driven by PAS- and CPE-mutated *Cnot6l* 3′-UTRs in GV oocytes. (**H** and **I**) Fluorescence microscopy (H) and western blot (I) results showing the translation levels of Flag-GFP driven by PAS1-mutated *Cnot6l* 3′-UTRs in oocytes arrested at the GV stage and those developed to the MII stage.

To save space, translational activities of 3′-UTR reporters quantified by comparing the GFP and mCherry fluorescence intensities in Figures 3–6 were presented in Supplementary Figure S2.

### Efficiency of CPE-mediated translation inhibition was affected by the locations of CPEs relative to PAS

We evaluated whether the 4 CPEs in *Cpeb1* 3′-UTR repressed PAS2 with equal efficiency. When two CPEs located on the same side of PAS2 were mutated (ΔCPE1/2), PAS2-mediated translation was partially de-repressed. In contrast, when 2 CPEs flanking PAS2 were mutated (ΔCPE2/4), the de-repression effect was marginal (Figure 4A and B). From this observation, we concluded that when multiple CPEs were present, they repress PASs more effectively by flanking them rather than adjacent to them on only one side.

Next, we tested whether CPEs influence the function of PAS1. Mutation of four CPEs did not affect the translation of Flag-*Gfp*-3′-UTR_m_*_Cpeb1_*-ΔPAS2/3 at both GV and MII stages (Figure 4C and D). This might be due to the distance between PAS1 and its downstream CPEs. To confirm this hypothesis, we made a series of nucleotide deletions between PAS1 and CPE1. When 50 bp nucleotides remained between PAS1 and CPE1, the CPEs still did not affect the translation of Flag-GFP (Figure 4E and F). On the other hand, when the distance between PAS1 and CPE1 was further shortened to 35 and 15 bp, the PAS1-mediated translation of Flag-GFP was impaired (Figure 4E and F). As an alternative approach, insertion of an additional CPE at 35 bp downstream of PAS1 remarkably inhibited the translation of Flag-*Gfp*-3′-UTR_m_*_Cpeb1_*-ΔPAS2/3 (Supplementary Figure S3). In contrast, no inhibitory effect was observed when the CPE was inserted at 50 bp downstream of PAS1 (Supplementary Figure S3). Collectively, these results demonstrated that the CPEs only repressed mRNA translation when they were relatively close to PASs.

In addition to PASs and CPEs, a consensus Pumilio-binding element (PBE) is present in the middle region of *Cpeb1* 3′-UTR. It was reported in *Xenopus* that the presence of at least one PBE is required for translational activation of *Ccnb1* transcripts (16,39). However, deletion of this PBE does not prevent the meiotic resumption-coupled increase of *Cpeb1* 3′-UTR translational activity (Supplementary Figure S4).

### CPEs within the 3′-UTR of *Btg4* were required for translation inhibition at the GV stage

Different from *Cpeb1, Btg4* mRNAs accumulate in mouse oocytes at the GV stage but do not translate into proteins until meiotic resumption. Therefore, we employed *Btg4* as an example to further investigate the mRNA translational repression in GV oocyte. Similar to the study of *Cpeb1*, we cloned the 3′-UTR of mouse *Btg4*, which contains two PASs flanked by three CPEs (Figure 5A), to construct the pRK5-Flag-*Gfp*-3′-UTR_m_*_Btg4_* plasmid. The *in vitro*-transcribed Flag-*Gfp*-3′-UTR_m_*_Btg4_* mRNA was not translated after it was microinjected into GV stage-arrested oocytes cultured in medium containing milrinone (Figure 5B and C). This was consistent with the translational pattern of the endogenous *Btg4* mRNA (5). Previous results have shown that PAS1 and PAS2 in *Btg4* 3′-UTR were redundant for *Btg4* translation because they were close to each other (29). Mutant of all three CPEs resulted in de-repression of *Btg4* 3′-UTR translational activity at the GV stage (Figure 5B and C). The results of PAT assay also indicated an increased polyadenylation of Flag-*Gfp*-3′-UTR_m_*_Btg4_* mRNA after CPE mutations (Figure 5D). CPE mutations also facilitated the binding of CPSF4 with *Btg4* 3′-UTR, as detected by the RIP assay (Figure 5E).

Next, we evaluated the contribution of each CPE to the translational repression of the *Btg4* 3′-UTR. Mutations in CPE1 or CPE2 only caused marginal de-repression because the PASs were still flanked and repressed by the two WT CPEs (Figure 5F and G). In contrast, mutations in CPE3 caused a more significant de-repression. Consistent with our observations in *Cpeb1* 3′-UTR, this result indicated that the CPEs repressed translation more efficiently when flanking the PAS than when it was located on the same side of the PAS. Mutating two CPEs (ΔCPE1/3 or ΔCPE1/2) led to further de-repression of *Btg4* 3′-UTR, but the extent was still less than that due to the triple CPE deletion (ΔCPE1/2/3), indicating that CPEs inhibited translation in GV oocyte in a dose-dependent manner.

### The translation of *Cnot6l* at the GV stage was mediated mainly by the proximal PAS1

The results described above suggested that *Cpeb1* mRNA was translated in GV oocytes, but *Btg4* mRNA was not because all PASs of *Btg4* 3′-UTR were repressed by adjacent CPEs, while the proximal and distal PASs in *Cpeb1* 3′-UTR were exempted from CPE-mediated repression due to a distance effect. To further test if this hypothesis was widely applicable among oocyte transcripts, we employed the 3′-UTR of mouse *Cnot6l* (3′-UTR_m_*_Cnot6l_*) as the third example, for reasons described below.

A previous study reported that *Cnot6l* 3′-UTR was translationally dormant in GV oocytes and was activated after meiotic resumption (40). In the study by Ma *et al.* (40), the authors used a truncated 3′-UTR fragment (402 bp, which contained three CPEs and a distal PAS closely adjacent to the last CPE) in the reporter experiment. We have noticed that there was an additional PAS and CPE in the proximal region of *Cnot6l* 3′-UTR, which was not included in the fragment cloned by Ma *et al.* (40) (Figure 6A). Therefore, we used the 1597 bp *Cnot6l* 3′-UTR fragment containing two PASs and four CPEs in our reporter experiment, and obtained different results.

We *in vitro*-transcribed the mRNA encoding Flag-*Gfp*-3′-UTR_m_*_Cnot6l_* and microinjected it into GV oocytes. The fluorescence and western blot results showed that the FLAG-GFP reporter was translated in GV oocytes (Figure 6B and C). The PAS1 mutation almost completely abolished the translational activity of *Cnot6l* 3′-UTR at the GV stage (Figure 6B and C). On the other hand, the PAS2 mutation did not affect *Cnot6l* 3′-UTR activity at the GV stage, suggesting that PAS2 was repressed by CPEs and the *Cnot6l* 3′-UTR activity at the GV stage was solely mediated by PAS1 (Figure 6B and C). The results of PAT assay verified that mutations in PAS1, but not PAS2, in the *Cnot6l* 3′-UTR abolished tail polyadenylation at the distal end (Figure 6D and E).

**Figure 7.**
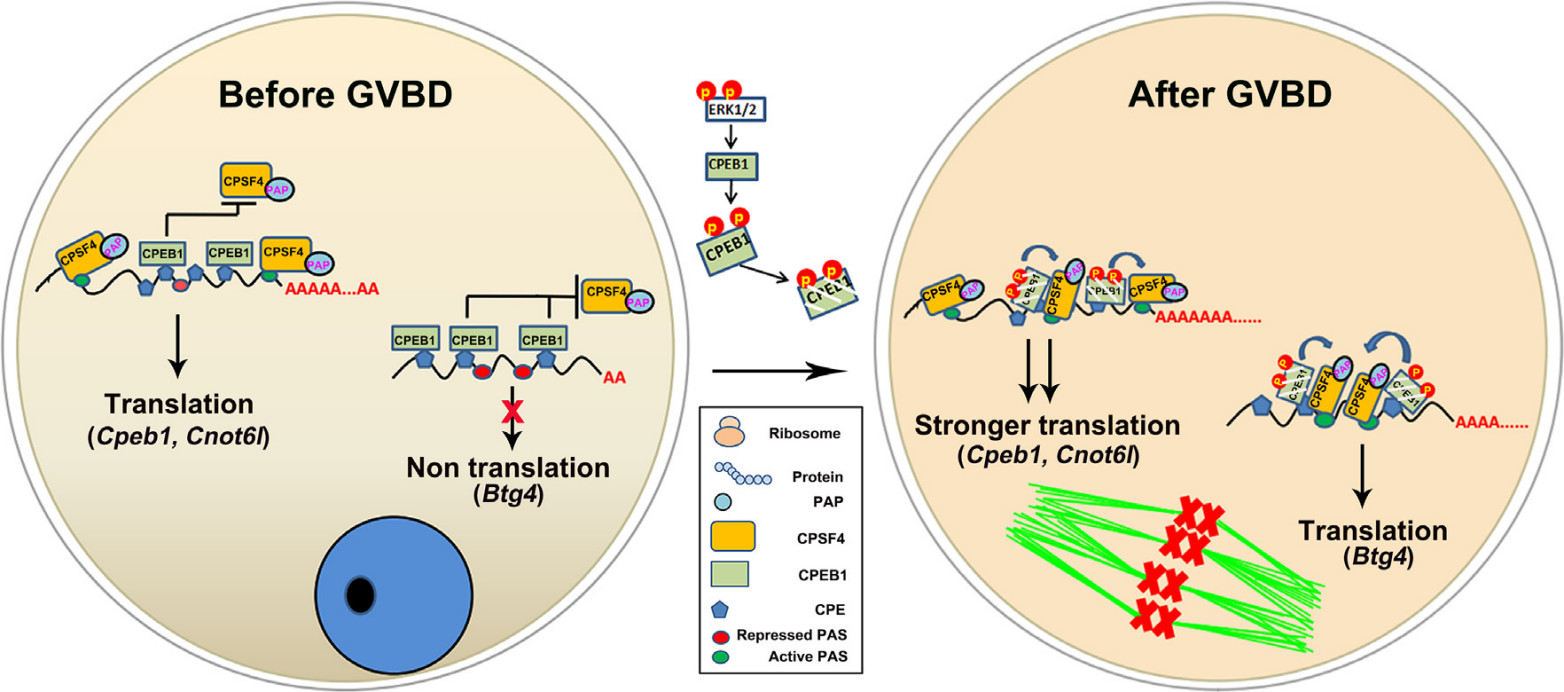
Models that PASs and CPEs regulates the mRNA translation in oocytes. In oocytes at the GV stage, some transcripts (*Cpeb1* and *Cnot6l*, for example) are translationally active because PASs that are distant from CPEs recruit CPSF4 and mediate the translation. Meanwhile, some other transcripts (*Btg4* for example) are translationally dormant because all PASs in their 3′-UTRs are repressed by surrounding CPEs, which recruit CPEB1 proteins and prevent the binding of CPSF4 with the PAS. In maturing oocytes after GVBD, CPEB1 proteins are phosphorylated and partially degraded by the meiotic resumption-coupled MAPK cascade, and are converted from translational inhibitory factors to stimulating factors. All PASs become accessible to CPSF4. As a result, the dormant transcripts (such as *Btg4*) are actively translated, and translation of active transcripts (such as *Cpeb1* and *Cnot6l*) are strengthened due to the activation of additional PASs.

Furthermore, mutations in CPE4, which was closely adjacent to PAS2, restored PAS2-mediated translation. On the other hand, mutations in CPE2 and 3, which were further away from PAS2, failed to relieve the PAS2 from its dormant state (Figure 6F and G). Although the PAS1 mutated *Cnot6l* 3′-UTR was not translationally active in GV oocytes, its translational activity was restored after meiotic resumption (Figure 6H and I). Mutation of CPE4 or RNAi depletion of endogenous CPEB1 significantly increased the accessibility of PAS2 to CPSF4 proteins (Supplementary Figure S5), confirming that CPEB1 binding to CPE4 was responsible for masking CPSF binding to PAS2. These results further proved our hypothesis that CPEs only repressed translation when they were located closely to a PAS in the 3′-UTR, and were converted from translational repressing elements to translational stimulating elements (Figure 7).

## DISCUSSION

The maternal mRNAs stored in fully grown mammalian oocytes contain short poly(A) tails, and it is only when these tails are elongated that translation can occur (14,41). Cytoplasmic polyadenylation requires two elements in the 3′-UTR, the PAS and the CPE, which also participates in the translation repression of its target transcripts in a quiescent state (16). However, not all CPE-containing mRNAs are repressed or activated at the same time during the meiotic cell cycle, and polyadenylation is temporally and spatially regulated in oocytes (39). In previous studies using *X*. oocytes, a set of rules have been postulated that can be used to predict the translational behavior of CPE-containing mRNAs during meiotic maturation (16,39). We compared those results with our current study, and found several features that were not described before as well as regulatory rules specific for mammalian species. These include the following:

- Previous models suggest that the PAS at the end of the 3′-UTR was responsible for cytoplasmic polyadenylation and translation (42). Our data showed that both the proximal and distal PASs were able to mediate cytoplasmic polyadenylation and translation of maternal transcripts, as long as they were not repressed by adjacent CPEs. Furthermore, we provided evidence that this proximal PAS-mediated non-canonical cytoplasmic polyadenylation depends on recruitment of CPSF to the PAS. Nonetheless, it remains unclear whether this non-canonical polyadenylation mechanism only functions in the ooplasm or also widely exists in the nuclear polyadenylation process of somatic cells (43).
- Previous studies only investigated the combinatorial code that determined meiotic maturation-coupled mRNA translation, but not the transcripts that were constitutively translated in GV stage-arrested oocytes (39). Instead, we deciphered the combinatorial code that determined meiotic maturation-independent mRNA translation: at least one PAS located relatively far away (> 35-50 bp) from the CPEs was essential for the exemption of translational repression in GV oocytes (Figure 7). Importantly, this PAS does not have to be at the end of the 3′-UTR, due to the noncanonical polyadenylation mechanism we identified.
- Results in *X*. oocytes showed that translational repression requires a cluster of at least two CPEs, irrespective of its position along the 3′-UTR (16). In contrast, the data of our study in mouse oocytes indicated that a single CPE was sufficient for efficient translational repression as long as it was located close enough to the PAS. Furthermore, even for the 3′-UTR containing a cluster of CPEs, their relative positions to the PAS were crucial for the repressing effect.
- In *X*. oocytes, translational activation requires at least a single CPE together with a PBE (16,24). Nevertheless, deletion of the single PBE in the *Cpeb1* 3′-UTR did not prevent translational activation. Furthermore, the 3′-UTRs of *Btg4* and *Cnot6l* do not contain PBEs, but we still observed CPE-dependent translational activation during meiotic maturation, indicating that PBEs were not required for CPE-mediated translational activation in mouse oocytes. This conclusion was supported by the studies in oocyte-specific *Pum1/2* knockout mice (44). Although these female mice showed reduced fertility, the defects of meiotic maturation in *Pum1/2*-knockout oocytes were rather moderate, suggesting that the role of mammalian PUM in translational regulation was not as essential as its *Xenopus* and *Drosophila* homologs.

To determine the rules of translational regulation in mammalian oocytes, a few new techniques and modified experimental approaches were used in this study:

- A nested PAT assay was employed to avoid amplifying the 3′-UTR of endogenous transcripts when we assessed the role of a given PAS or CPE (33). Furthermore, using this method, we selectively amplified the 3′-terminus of the full-length transcripts to measure the extent of polyadenylation. Therefore, we rule out a possibility that the observed PAS was contributed by some truncated or partially degraded transcripts.
- In previous studies in *Xenopus* and mouse oocytes, a luciferase assay-based reporter system was used to evaluate 3′-UTR activities (16,25). On average, three to five mouse oocytes were pooled and lysed as a sample for detection on a luminometer. To improve the analyses of 3′-UTR activities in oocytes, we generated a series of GFP reporter plasmids to evaluate the translational activity of the given 3′-UTRs (4,5). The accumulation of GFP signal driven by the 3′-UTRs fused with *Gfp* cDNA was not only visible by eye in each oocyte, but also feasible for quantification by comparing its fluorescence intensity with the signal of a constitutively translated mCherry protein. Furthermore, the Flag-tagged GFP proteins can be easily detected by western blotting (50 oocytes per sample), such that we were able to compare the translation activities of different 3′-UTRs on the same X-ray film. Another advantage of this approach was that the actual cell cycle stage of the oocytes can be determined by blotting for endogenous cell cycle marker proteins such as phosphorylated ERK1/2.
- Only the 3′-UTR of cyclin B transcripts were employed to investigate the combinatorial code of cytoplasmic polyadenylation in *Xenopus* (16). Therefore, how the rules summarized from these results are representative for other transcripts remain unknown. Instead, we analyzed the 3′-UTRs of three different genes: *Cpeb1, Btg4* and *Cnot6l*. They were functionally connected, and have distinct translation dynamics during mouse oocyte maturation. *Cpeb1* was constitutively translated; its protein products repressed translation of other transcripts at the GV stage including *Btg4* and *Cnot6l* (15,22). *Btg4* was dormant at the GV stage, but was actively translated after meiotic resumption (6,29); *Cnot6l* encodes a CCR4–NOT catalytic subunit. Accumulation of BTG4 and CNOT6L was crucial for triggering the timely mRNA decay and MZT, therefore forming an irreversible negative feedback that drove meiotic cell cycle progression (5,40). This strategy of dissecting the 3′-UTR code using gene sets instead of a single gene is an advantage of this study that has not been reported before.

In this study and previous reports, we have confirmed the presence of the endogenous full-length *Cpeb1, Btg4* and *Cnot6l* transcripts, which contained the same 3′-UTR fragments we used for reporter assays (4,5,40). For *Btg4* and *Cnot6l*, the 3′-UTR reporters showed similar patterns of polyadenylation and protein translation as the endogenous transcripts. In contrast, the regulation of CPEB1 protein levels in maturing oocytes was more complicated. Both *Cpeb1* mRNAs and proteins were abundantly expressed in GV-arrested oocytes. Upon oocyte meiotic resumption, ERK1/2 was activated by upstream kinases and triggered CPEB1 phosphorylation on Ser-184 and Ser-207 as well as CRL1^βTrCP^-dependent degradation (4). The phosphorylation and partial degradation of CPEB1 (70–90%) cause a change in the CPEB/CPE ratio and stimulated polyadenylation and translational activation of maternal mRNAs including *Cnot6l* and *Btg4*. Therefore, although we observed an increased translational activity of *Cpeb1* 3′-UTR after GVBD, the endogenous CPEB1 protein level decreased, instead of increasing, due to the additional layer of regulation on CPEB1 protein stability. This discrepancy does not affect the interpretation of our result where CPEs switched from repressing PAS2 to stimulating its translational activity following meiotic resumption.

Although our results demonstrated that CPEs only repressed mRNA translation when they were relatively close to PASs, the actual distance between CPEs and PASs that is required to cause a repressing effect varied among different 3′-UTRs. For example, we have experimentally confirmed that a downstream CPE can only effectively repress the translation activity of PAS1 in *Cpeb1* 3′-UTR within 35–50 bp. On the other hand, the two PASs *in Btg4* 3′-UTR were strongly repressed by the upstream and downstream CPEs, which were 66 bp away from the PASs. It is conceivable that in addition to the linear genetic information within the mRNA sequences, the 3D structures of the mRNA 3′-UTRs must also play a role in translational repression or activation (27). Mechanistic studies in *X*. oocytes indicate that CPEB1 is a dual function RNA-BP that, when not phosphorylated, recruits poly(A) RNase (PARN) to deadenylate and repress maternal mRNAs (27). CPEB1 also mediates translational repression (masking) of maternal mRNAs in unstimulated oocytes by recruiting Maskin which prevents the binding of CPSF to PASs in transcripts (27). However, potential functions of Maskin-like proteins in mammalian oocytes has not been investigated.

Furthermore, other 3′-UTR binding factors that have been originally reported in *Drosophila*, including Smaug, Musashi and Pumilio, are also involved in cytoplasmic translational regulation (7,45). The physiological importance of these factors in mice have also been reported (44,46,47), but their roles in forming a more sophisticated combinatorial code that regulates 3′-UTR repression and activation need to be deciphered in future studies.

## Supplementary Material

Supplementary Data
