# Supplementary material for: A combinatorial code for mRNA 3′-UTR-mediated translational control in the mouse oocyte
Source: Nucleic Acids Res. 2018 Oct 18;47(1):328–40. doi: 10.1093/nar/gky971 (PMC6326793; doi:10.1093/nar/gky971)
Supplement: Supplementary Data [file gky971_supplemental_files.pdf]

Supplementary Information

Supplementary Figures

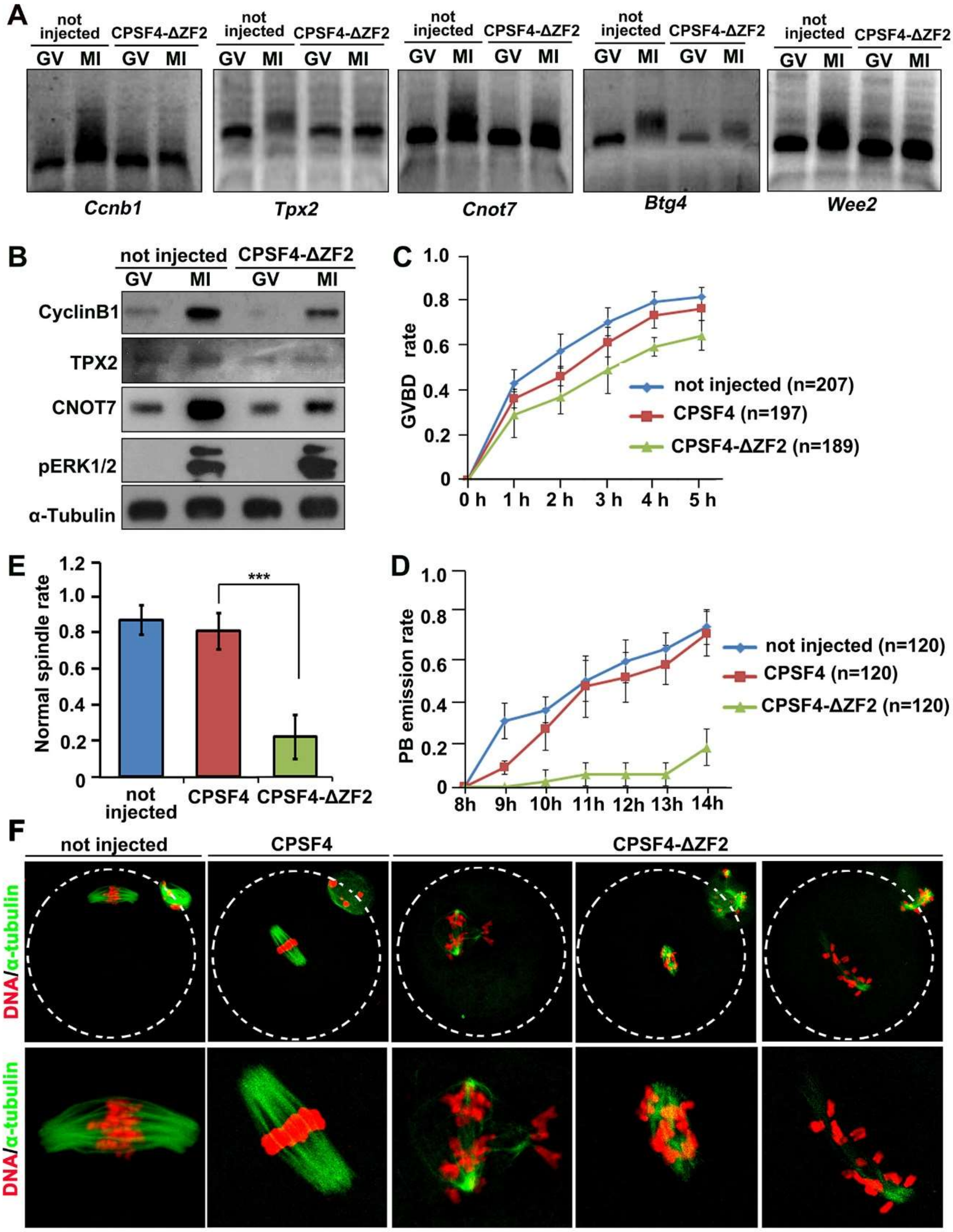

Supplementary Figure S1: Overexpression of a dominant negative form of CPSF4 blocked cytoplasmic mRNA polyadenylation and oocyte maturation.

A: Results of the PAT assay showing poly(A) tail lengths of the indicated transcripts with or without mRNAs expressing CPSF4-ΔZF2 being microinjected into oocytes at the GV stage. **B:** Western blot results showing levels of indicated proteins in GV and MI oocytes with or without microinjection of mRNA expressing CPSF4-ΔZF2. Endogenous α-tubulin was used as a loading control. Total proteins from 100 oocytes were loaded in each lane. **C-D:** Rates of germinal vesicle breakdown (GVBD) and polar body-1 emission (PBE) in oocytes microinjected with mRNAs encoding wild-type or ZF2 domain-deleted CPSF4 (CPSF4-ΔZF2). Error bars, s.e.m. The numbers of analyzed oocytes were indicated (n). **E:** Rates of oocytes containing a spindle of normal morphology at 16 h after mRNA microinjection as in **(F)**. Error bars, s.e.m. Statistically significant values of $P < 0.001$ by two-tailed Student's *t*-test is indicated by asterisks (***). **F:** Confocal microscopic results showing spindle assembly in oocytes microinjected with mRNAs encoding CPSF4 or CPSF4-ΔZF2. Images were taken at 16 h after mRNA microinjection.

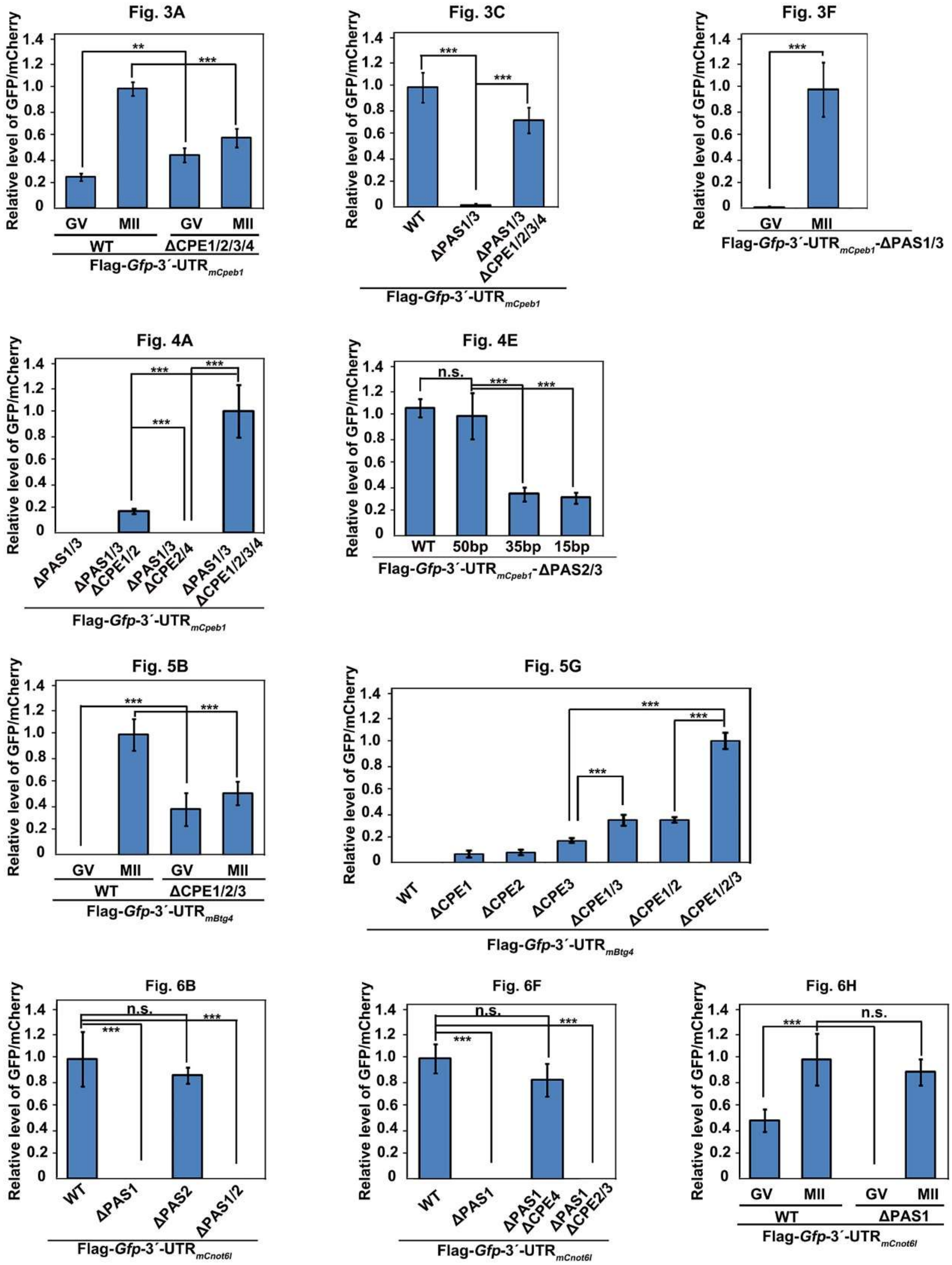

Supplementary Figure S2: Quantification of fluorescent signals in 3′-UTR reporter experiments.

Relative fluorescence intensity of GFP relative to mCherry in indicated figure panels were presented. Statistically significant values of $P < 0.05$, $P < 0.01$, and $P < 0.001$ by two-tailed Student’s *t*-test are indicated by asterisks (*), (**), and (***), respectively. n.s.: non-significant.

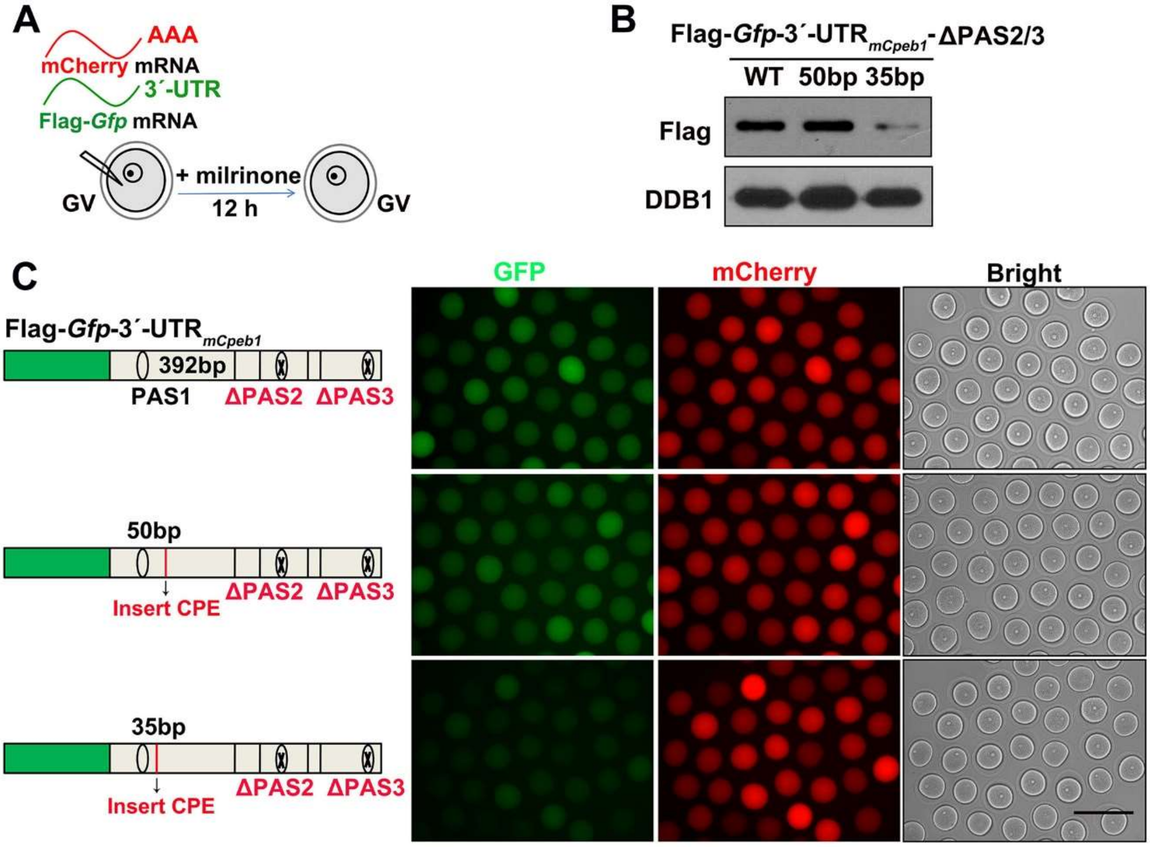

Supplementary Figure S3: Translation-repressing effect of CPEs) is determined by its distance to the PAS.

A: Illustration of mRNA microinjection and oocyte culture in **(B–C)**. **B–C:** Western blotting (**B**) and fluorescence microscopy (**C**) results showing expression level of Flag-GFP after PAS2/3 mutations and a CPE insertion in the *Cpeb1* 3′-UTR. Endogenous DDB1 was used as a loading control. Total proteins from 60 oocytes were loaded in each lane. Scale bar, 100 μm for all images.

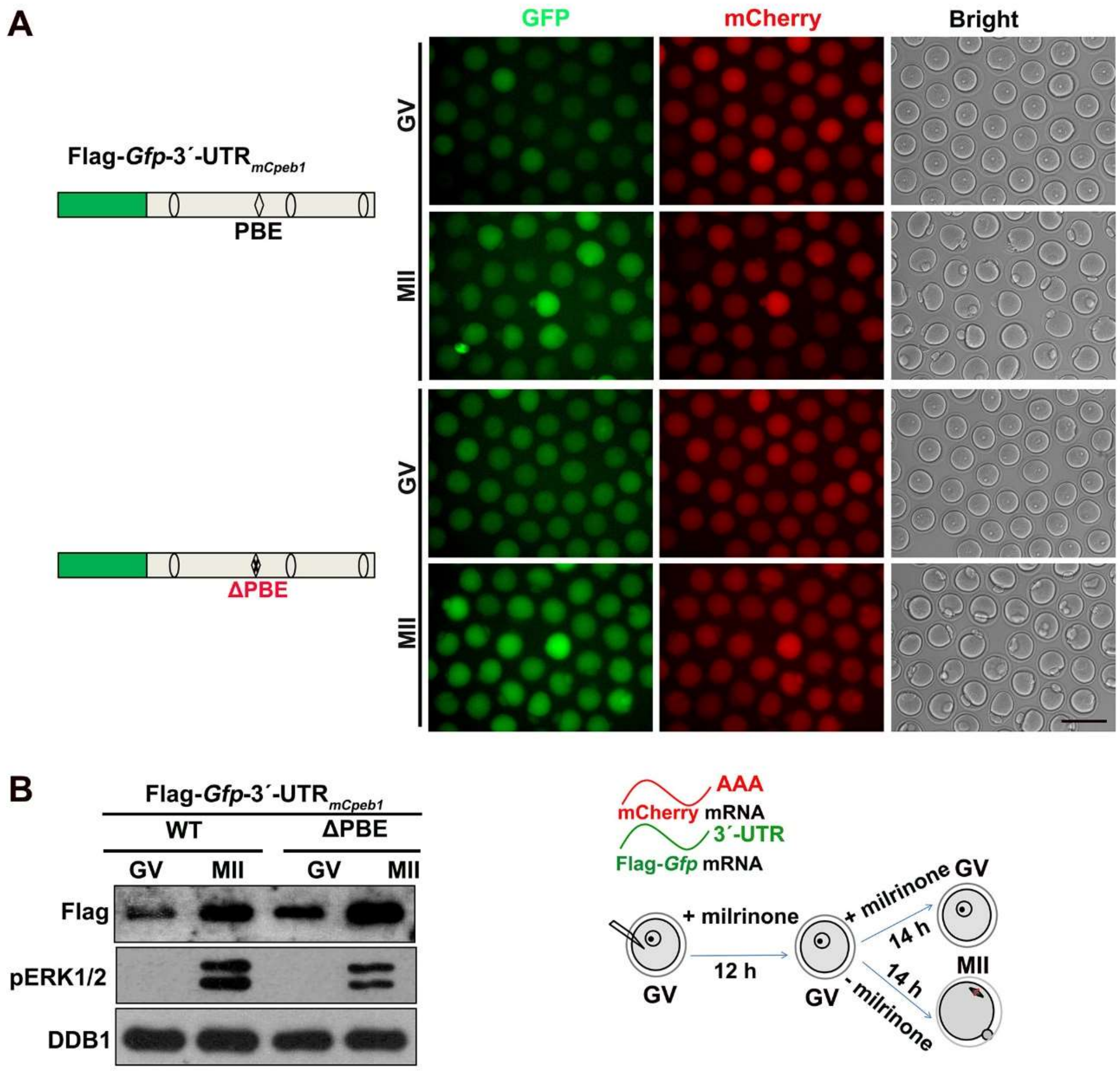

Supplementary Figure S4: Contributions of the Pumilio-binding element (PBE)to the translation activity of *Cpeb1* 3′-UTR in mouse oocytes.

A–B: Fluorescence microscopy (**A**) and western blotting (**B**) results showing the expression of Flag-GFP fused with *Cpeb1*3′-UTR or its PBE-mutated (ΔPBE) forms. Endogenous DDB1 was used as a loading control. Total proteins from 60 oocytes were loaded in each lane. Scale bar,100 μm for all images.

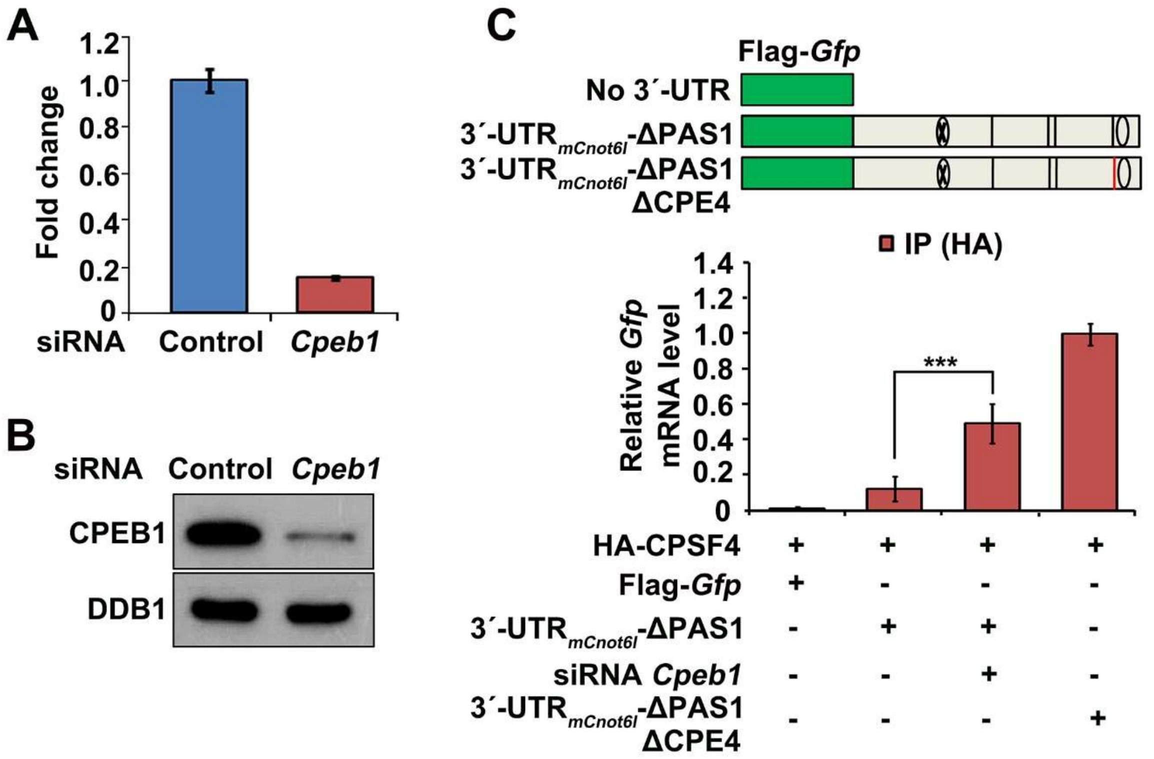

Supplementary Figure S5: Involvement of CPEB1 in CPE4-mediated translational repression of PAS2 in *Cnot6l* 3′-UTR.

A–**B:** Quantitative RT-PCR **(A)** and western blotting **(B)** results showing the deletion effect of *Cpeb1* siRNA. **C:** Ribonucleoprotein immunoprecipitation assay results using a HA antibody showing the interactions between HA-CPSF4 and indicated transcripts in GV oocytes. ***, $P < 0.001$ by two-tailed Student's *t*-tests. Levels of *Gfp* mRNA co-precipitated with CPSF4 was detected by quantitative RT-PCR.

Supplementary Tables

Supplementary Table S1. Primer sequences.

**Primer name**	**Target Gene**	**Application**	**Sequences (5'-3')**
R1	N.A.	Anchor primer for PAT assay	5'-GCGAGCTCCGCGGCCGCGTTTTTTTTTTT-3'
1st PCR-F	*Cpeb1*	PAT assay (with R1)	5'-CACCCAGTCCGCCCTGAGCAAAG-3'
2nd PCR-F			5'-TACTGGAATTGAAAACTTGACT-3'
Cpeb1-F	*Cpeb1*	PAT assay (with R1)	5'-CGCCACTCCTGTCTTGTAATGCCA-3'
Gfp-F	*Gfp*	RIP assay (Real-time PCR)	5'-CGCTACCCCGACCACATGAA-3'
Gfp-R			5'-CTTCAGCTCGATGCGGTTCA-3'
1st PCR-F	*Btg4*	PAT assay (with R1)	5'-CACCCAGTCCGCCCTGAGCAAAG-3'
2nd PCR-F			5'-CACTAGTGAATATAGGTGCCTAGA -3'
1st PCR-F	*Cnot6l*	PAT assay (with R1)	5'-CACCCAGTCCGCCCTGAGCAAAG-3'
2nd PCR-F			5'-GAGAAAATTAGTTGGCAAAATTA-3'
Ccnb1-F	*Ccnb1*	PAT assay (with R1)	5'-CGCCACTCCTGTCTTGTAATGCCA-3'
Tpx2-F	*Tpx2*	PAT assay (with R1)	5'-CTGGAAGTCAGTCTTCCACTAG-3'
Cnot7-F	*Cnot7*	PAT assay (with R1)	5'-TGGACTACAAGTTGTAATGTGTG-3'
Btg4-F	*Btg4*	PAT assay (with R1)	5'- GTAGGTTTTCAACTAAGGAAGAT-3'
Wee2-F	*Wee2*	PAT assay (with R1)	5'-GGCAGAGGACCCAGGAATTTTG-3'
Cpsf4-F	*Cpsf4*	Real-time PCR	5'-GCAAGAAAGGGGACCAGTGTGAGT-3'
Cpsf4-R			5'-TACAGAAGCCGCGGTCATACCAAG-3'
Cpeb1-F	*Cpeb1*	Real-time PCR	5'-GGACCTTCTTGGAGCTCCTA-3'
Cpeb1-R			5'-GAACGCCTCCTAGGAACACC-3'

Supplementary Table S2. Antibody information.

Protein name	Manufacture (catalogue number)	Applications (working dilution)	Website Link
**FITC-α-Tubulin**	Sigma (F2168)	WB (1:1000)<br>IF (1:500)	http://www.sigmaaldrich.com/catalog/product/sigma/f2168?lang=zh&region=CN
**ERK1/2**	Santa Cruz (sc-94)	WB (1:1000)	http://www.scbt.com/datasheet-94-erk-1-k-23-antibody.html
**pERK1/2**	Cell Signaling (9101)	IF (1:400)	http://www.cellsignal.com/products/primary-antibodies/phosphop44-42-mapk-erk1-2-thr202-tyr204-antibody/9101
**FLAG**	Sigma (F3165)	WB (1:3000)	http://www.sigmaaldrich.com/catalog/product/sigma/f3165?lang=zh&region=CN
**HA**	Cell Signaling (3724)	WB (1:2000)	http://www.cellsignal.com/products/primary-antibodies/ha-tagc29f4-rabbit-mab/3724
**DDB1**	Epitomics (3821-1)	WB (1:1000)	http://www.epitomics.com/products/search/DDB1
**CPSF4**	Proteintech (15023-1-AP)	IF (1:200)<br>WB (1:1000)	https://www.ptglab.com/results?q=cpsf4
**CPEB1**	Proteintech (13274-1-AP)	WB (1:500)	https://www.ptglab.com/products/CPEB1-Antibody-13274-1-AP.htm
